# Innate lymphoid cells—Underexplored guardians of immunity

**DOI:** 10.1371/journal.ppat.1011678

**Published:** 2023-10-19

**Authors:** Irina Tsymala, Karl Kuchler

**Affiliations:** Department of Medical Biochemistry, Max Perutz Labs Vienna, Medical University of Vienna, Campus Vienna Biocenter, Vienna, Austria; University of Maryland, Baltimore, UNITED STATES

## Introduction

Innate lymphoid cells (ILCs) show remarkable plasticity and influence immunity as early sensors of pathogenic cues [1–4]. The general term “ILC” describes innate immune cells that display most common features of lymphocytes. ILCs comprise 5 different subsets: cytotoxic natural killer cells (NK cells), lymphoid tissue inducer cells (LTi), and 3 helper-like subsets, group 1 ILCs (ILC1), group 2 ILCs (ILC2), and group 3 ILCs (ILC3) [3,5–7]. ILCs are defined by their specific cytokine and transcription factor profiles, largely mirroring functions of T helper (Th) subsets [3,4,8]. However, the fate of ILCs is determined by Id2, which blocks T cell differentiation and drives ILC development [2,9,10]. As a consequence, ILCs typically lack antigen-specific T or B cell receptors [3], sandwiching their roles in between adaptive and innate immunity. In steady-state, ILC1s depend on T-bet and express interferon gamma (IFN-γ), as well as tumor necrosis factor alpha (TNF-α). ILC2s engage GATA3, RORα, Bcl11b along with GFI1 upon differentiation [2,3] and produce IL-13, IL-5, IL-4, IL-9, and amphiregulin [2,11,12]. Finally, ILC3s require RORγt, as well as AhR while secreting IL-22 and IL-17 [4,13–15].

Most ILCs are long-lived, self-renewing cells residing at tissue barriers, were they readily respond to physiological or pathological triggers by cytokine release [2,4,16,17] (Table 1). Thereby, ILCs may acquire long-lasting immunological memories relevant for effective immune surveillance [18–21]. ILCs can also fuel dysregulated immune responses causing chronic inflammation and thus worsen disease outcomes [4,22,23] (Table 1). However, biological mechanisms modulating polarization of ILCs in response to pathogens remain ill-posed, and even less is known about the plasticity between NK cells and ILCs [4,24]. Further, considerable differences between human and murine ILCs have been posing road blocks to gain a better understanding of their immunological functions [25,26]. In this work, we provide an overview about the properties of main ILC subsets, emphasizing possible roles in infectious diseases. We will discuss plasticity in response to different pathogens. Finally, we shall inspect the immunomodulatory potential of ILCs in patients, as well as future challenges in ILC biology.

**Table 1:** Relevance, functions, and regulation of ILCs in infectious diseases.

Tissue	Pathogen	ILC subset	Relevance in infection
**Intestine**	*C*. *jejuni*	**ILC1**	• Colitogenic NK1.1^-^ IFN-γ^+^ ex-ILC3s [23]
**Intestine**	*C*. *difficile*	**ILC1, ILC3**	• ILC1s and ILC3s cooperate in host defence [97]
**Intestine** **Liver**	*S*. Typhimurium [12,40,61] *L*. *monocytogenes* [40]	**ILC1, ILC3**	• Protective IFN-γ^+^ NKp46^+^ ILCs [40,59] • RORα regulates late pro-fibrotic IL-17A secretion [12] • ThPOK maintains IFN-γ release in NKp46^+^ ILCs [59] • Runx3 triggers protective IFN-γ secretion [40]
**Intestine** **systemic**	*T*. *gondii*	**ILC1**	• Protective TNF-α^+^ IFN-γ^+^ ILC1s [18,98] • Formation of memory Ly6c^+^ ILC1s [18]
**Intestine**	*C*. *rodentium*	**ILC3, LTi**	• Trained IL-22^+^ILC3 protective [99] • IL-22^+^ILCs critical in early phase [54,55,100–104] • GM-CSF^+^NKp46^+^ILC3s redundant for clearance [54] • IL-22^+^Nkp46^+^ maintain caecal homeostasis [103] • IL-22^+^ T_H_ cells mediate clearance [54,55,103,104] • Vitamin D impacts IL-22 response [101]
**Liver**	Cytomegalovirus	**ILC1**	• Cytotoxic Ly49E^+^ ILC1s protective [92] • Early—protective IFN-γ^+^ ILCs [38,92] • m12-driven protective response (re-challenge) [19]
**Oral mucosa**	Vaccinia virus	**ILC1**	• ILC1s are priming antiviral response via IFN-γ [39]
**Oral mucosa**	*C*. *albicans*	**ILC3, iILC2**	• Protective, early IL-17 secretion [43,57]
**Lung**	*A*. *fumigatus*	**ILC2**	• Induce tissue hyperreactivity in cystic fibrosis [14]
**Lung**	*C*. *neoformans*	**ILC2**	• ILC2s induce tissue hyperreactivity [22]
**Lung** **intestine**	*N*. *brasiliensis* *T*. *muris* [46]	**ILC2**	• Protective (MHCII^+^) ILC2 response [46,68,105] • Areg secretion by ILC2s essential for resistance [46] • T cells required for clearance [41,68] • ILC2s secrete IL-5 constitutively [41] • IL-33, IL-25, and TSLP activate ILC2s and T_H_2s [41]
**Lung**	*M*. *tuberculosis*	**ILC1, ILC3**	• Protective “trained” ILC1-like cells [20,34] • Protective CXCR5^+^ ILC3s [95]
**Lung**	*S*. *pneumoniae* [56,106] *K*. *pneumoniae* [107] *P*. *aeruginosa* [108]	**ILC3**	• IGF1 instructs ILC3 development [106] • IL-17A^+^ILC3s recruit Ly6c^hi^ monocytes [107] • IL-22^+^ ILC3s improve survival [56,106,108]
**Lung**	Influenza virus A	**ILC1, ILC2**	• ILC2s contribute to wound healing [93,109] • Areg supports tissue integrity [109] • ILC1s support viral clearance [28,110] • IL-5 and IL-13 promote inflammation [111]

ILC, innate lymphoid cell; IFN-γ, interferon gamma; LTi, lymphoid tissue inducer cells; NK, natural killer; TNF-α, tumor necrosis factor alpha; TSLP, thymic stromal lymphopoietin.

## NK cells and ILC1s—Two sides of the same coin?

**Fig 1:**
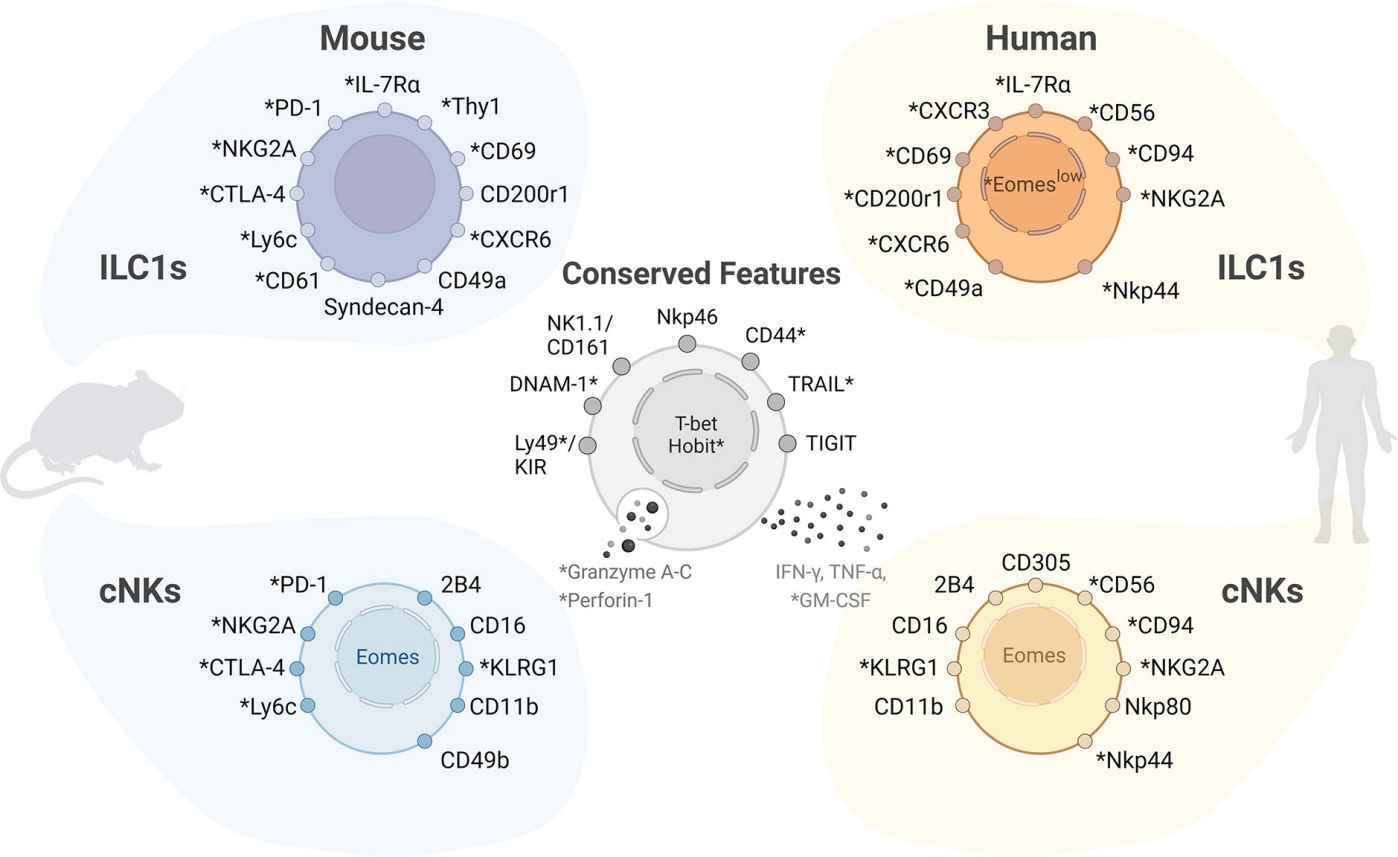
ILC1s and conventional NK cells (cNKs) express common and distinct cell surface receptors. Both subsets share interspecific conserved features, including cell surface markers (Ly49-family receptors in mouse/Killer Cell Immunoglobulin like Receptors, KIRs, in human, DNAM-1, NK1.1 in mouse/CD161 in human, Nkp46, CD44, TRAIL, TIGIT), transcription factors (T-bet, Hobit), cytotoxic features (granzyme A, B and granzyme C in mouse, perforin expression), and cytokine expression profile (IFN-γ, TNF-α, GM-CSF). Mouse ILC1s can be distinguished by the lack of lineage-defining surface molecules (T or B cell receptor, CD19, B220) and the expression of CD200r1, Syndecan-4, CD49a, and IL-7Rα. Lineage-negative human ILC1s may express Eomes at low levels as well as surface receptors associated with cNK cells; (*)-marked features are only present in certain environments. Created with BioRender.com. cNK, conventional NK; ILC, innate lymphoid cell.

Although NK cells and ILC1s arise from a common lymphoid progenitor [2], both express IFN-γ and both are implicated in the early clearance of intracellular pathogens [27]. Especially human NK cells are phenotypically difficult to distinguish from ILC1s due to their overlapping surface receptor expression, such as NKp46, NK1.1 (mouse), CD56 (human) [6,27] (Fig 1), and owing to a lack of unique, subset-defining hallmarks.

The NK lineage commitment is initiated by Eomesodermin (Eomes), whereas ILC1 up-regulates T-bet instead, along with surface receptors such as IL-7Rα, CD90, CD49a, and CD200r1 [6,21,28,29] (Fig 1*)*. Expression of these subset markers may differ depending on their microenvironment and activation state, often resulting in a rather blurred distinction between tissue-resident NK cells and ILC1s [3,30–32]. Although NK cells and ILC1s diverge during development, some degree of plasticity may be maintained. Of note, TGF-β induces a metabolic transition in NK cells, promoting oxidative phosphorylation and adoption of an ILC1-like phenotype, again highlighting the close relation of these subsets [6,20].

ILC1s can be primed by infections that favor the acquisition of memory-like phenotypes [33]. Similar to memory T cells, pathogen-trained ILC1s show distinct transcriptional patterns [19,20]. Trained ILC1s can increase expression of Ly6c, IL-2Rα, IL-7Rα, and IL-18R and contribute to pathogen resistance after re-challenge by increasing IFN-γ secretion [18–20,34,35]. In a mouse model of pulmonary *Mycobacterium tuberculosis* (Mtb) infection, adoptive transfer of IL-18R^+^ IFN-γ-expressing ILCs improves pathogen clearance in lymphocyte-deficient mice [20]. A pathogen-experienced ILC1-like subset also appears after *Toxoplasma gondii* infections [18], since a Stat4-dependent ILC subset persists in the circulation resembling mature NK-cells by surface Ly6c [18,35].

Although ILC1s can produce cytolytic granules, their response mainly entails cytokine secretion and signaling [24,29]. Transition to a mature cytotoxic effector state in ILC1s results in the down-regulation of IL-7Rα regulated by Hobit (Homolog of Blimp-1 in T cells), similar to invariant natural killer T cells (iNKT) and NK cells [36]. Notably, ILC1s share key-effector properties with iNKTs and NK cells, while their physiological functions as cytotoxic effector cells remain unresolved. ILC1s may be dispensable for the control of intracellular pathogens if they are compensated for by other cytotoxic subsets, including conventional NK cells or cytotoxic T cells [37,38]. Nonetheless, IFN-γ secretion by ILC1s can impede viral replication in various mouse models [20,28,34,39,40]. ILC1s are mainly located at epithelial barrier sites and respond within the first hours of infection, acting even before cytotoxic NK cells. This fast-acting surveillance or danger-sensing may offer a crucial advantage to host defense [38,39].

## ILC2s—Indispensable for mucosal type-2-immunity?

ILC2s respond to mucosal alarmins such as IL-25, thymic stromal lymphopoietin (TSLP) as well as IL-33 [41]. Especially engagement of ST2 (IL-33R) triggers fast expansion of ILC2s [41–43]. Consequently, ILC2s amplify allergic response, eosinophilia, and hyperreactivities in the skin, lung, and respiratory tract.

ILC2s are also associated with excessive inflammation in mucosal infections [22,43]. For example, ILC2s exacerbate *Aspergillus fumigatus* infections in a mouse model of cystic fibrosis, where mast cells activate ILCs via IL-2 signaling, in turn resulting in IL-9 release by ILC2s and Th9 cells [14]. Furthermore, depletion of ILC2s leads to prolonged survival of mice infected by *Cryptococcus neoformans*, as type-1-immunity and pro-inflammatory activation of pulmonary macrophages is enhanced [22]. ILC2 expansion during inflammation is tightly linked to their distinct metabolic profile as defined by Arginase-1 (Arg1) expression [4,44]. Inhibition of Arg1 dampens ILC2s but not ILC3s, and affects several metabolic pathways, including aerobic glycolysis [4,44]. Further, *Mtb*-associated type-1-inflammation and reprograming towards glycolysis induces a conversion of ILC2s into IFN-γ^+^ ILC1-like cells [4,20]. In contrast, *in vitro* challenge with *Pseudomonas aeroginosa*, *Staphylococcus aureus*, or a combination of IL-23, IL-1β, and TGF-β induces transition towards an ILC3-like phenotype [45].

Of note, a targeted depletion of ILC subsets has been difficult to achieve, since they share common surface receptors and lineage-defining transcription factors with conventional NK or T cells. Interestingly, the recently identified neuromedin U receptor 1 (Nmur1) might form an exception, since ILC2s constitutively express Nmur1, which distinguishes them from other immune cells. Thus, ILC2s may also play a role in the cross-talk with sensory neurons [2,46,47]. Indeed, ectopic Nmur1 expression allows for the generation of mouse models suitable for *in vivo* tracking and targeted gene-deletion in ILC2s [46,47]. For instance, depletion of ILC2s, using Nmur1^iCre-eGFP^ Id2^fl/fl^ or Nmur1^iCre-eGFP^ Gata3^fl/fl^ mice, reveals that eosinophil infiltration during pulmonary helminth infections is directly linked to early IL-5 secretion by ILC2s [47]. In addition, ILC2-deficiency in Klrg1^cre^Gata3^fl/fl^ mice significantly delays complete clearance of intestinal *Nippostrongylus brasiliensis* infections [48]. Consequently, the presence of ILC2s is vital for clearance of helminth infections, as well as for inducing early type-2-inflammation [47,48]. Furthermore, ILC2s are a main source of amphiregulin in intestinal tissues, exerting a protective regulatory impact on mucosal epithelia in homeostasis but also during parasite infection [46]. ILC2s can also regulate inflammation via IL-10 secretion [3,49,50]. In contrast to T_reg_ cells, ILC2s do not require FoxP3 [51]. Instead, IL-10 induction in ILC2s is associated with the up-regulation of Id3, but also Atf3, Blimp-1, cMaf, Klf2, and Foxf1 may be involved [49–51]. Further, retinoic acid, IL-2, IL-10, IL-27, IL-4, and NMU can induce regulatory ILC2s *in vitro* while the TL1A functions as a negative regulator [50,51]. Although regulatory ILC2s are pivotal in allergy and tissue integrity, their relevance and potential roles during infections remain open [49,50].

## ILC3s—Threat or treat for intestinal immunity

RORγt guides the maturation of ILC3s and LTis, the latter being essential for the development of secondary lymphoid tissues [2,9]. LTis differ from ILC3s by their cell surface expression of CCR6, CD4, and c-Kit [2,52]. ILC3s inhabit the lamina propria of the small intestine and colon, as well as other mucosal tissues, where they respond to microbial and dietary compounds engaging the AhR-signaling pathway [2,4,52,53]. In contrast to Th cells, ILC3s develop also in germ-free mice [9].

Mucosal ILC3s secrete IL-17 and GM-CSF in the early phase of infections to recruit neutrophils and to promote their activation [2,5,54]. In addition, ILC3s-mediated cross-talk with epithelial cells via IL-22 sustains barrier integrity and supports the release of defensins and antimicrobial peptides to restrain excessive damage after infection [55,56]. Resident ILC3s, as well as recruited IL-17-producing RORγt^+^ ILC2s, are early sources of IL-17A, thus contributing to the control and perhaps clearance of mucosal candidiasis in absence of adaptive immunity [43,57]. Of note, dysregulated colonization of the gut with *Candida* spp. is instead associated with activation of ILC3s that display tumorgenic potential [58]. Further, IL-17A^+^ILC3s can also contribute to *Salmonella enterica* Typhimurium serovar (*S*. Typhimurium)-induced gut fibrosis [12]. IL-17A production at later stages of chronic disease is regulated by RORα and becomes a driving factor of gut fibrosis.

ILC3s can release IFN-γ after gradually up-regulating T-bet. Interestingly, these beneficial T-bet^+^ILC3s (ex-ILC3s) restrain *S*. Typhimurium as well as *Listeria monocytogenes* infections [40,59]. Contrary to IL-22^+^ ILC3s, ex-ILC3s are redundant in intestinal *Citrobacter rodentium* infections, and they promote inflammation via GM-CSF signaling [54,60,61].

ILC3s also cooperate with other lymphocytes to orchestrate intestinal immunity and to regulate the response to microbiota and pathogens during aging [17,62–64]. For instance, certain E3 ubiquitin ligases, such as cIAP1/2, modulate proliferation and function of γδ-T cells and intestinal ILC3s [64]. Defects in cIAP1/2 affect immunity only gradually over time, comprising ILC3-related IL-22 production and increasing susceptibility to *C*.*rodentium* infection.

A subset of ILC3s can also execute MHCII antigen presentation, and they express co-stimulatory surface molecules [63,65]. Although a few studies indicate that ILC3s can process and present antigens to T cells, this matter remains controversial [63,66,67]. A regulatory effect on T cell homeostasis may arise from competition for IL-2 recognition, albeit without providing co-stimulatory signals [63]. Of note, a novel extrathymic autoimmune regulator (AIRE) and MHCII-expressing cell lineage could mediate microbial-tolerance in early development. This subset appears related but transcriptionally distinct from dendritic cells and ILCs subsets [66,67].

Notably, MHCII-related functions are not restricted to ILC3-like polarization, as they are also observed in ILC2s and NK cells [68–70]. Moreover, a variety of other innate immune cells including NK cells and myeloid cells may have similar barrier-protective properties. Accordingly, ILC3s are important for barrier integrity, but their role in mucosal immune surveillance needs to be further elucidated.

## Can helper-ILCs exhibit immunomodulatory functions in patients?

Current knowledge about the identification and function of helper ILCs is largely based on mouse models. However, surface markers that separate ILCs clearly from other lineages, especially human ILC1s from NK cells, are still missing. Further, more clinical studies and disease implications will be critical to determine whether ILCs can offer targetable cell types in infectious disease settings. Engraftment of human ILC precursors into humanized or immunodeficient (NSG) mice may be a first step to explore the immunomodulatory potential of ILCs. This approach may also provide appropriate models to investigate the development, expansion, and plasticity of transferred ILC-precursors in the human setting [26,71,72]. The low abundance of peripheral blood ILCs pose extreme technical challenges for isolation and identification. Hence, robust methods for *ex vivo* expansion of ILC-progenitors from peripheral blood, bone marrow, or umbilical cord blood are urgently needed [42,71,73]. Of note, proof-of-concept studies show that human ILCs can be obtained from various sources, remain viable and functional under *in vitro* expansion conditions, offering interesting options for cell-based therapies similar to T or NK cells [73].

Importantly, haplo-identical and allogeneic NK cells are tested in several clinical studies [74–76]. NK cells can be engineered *in vitro* to enhance their CD16-mediated antibody-dependent cytotoxicity (ADCC) rendering them effective against hematopoietic malignancies [74]. Several strategies are currently used to enhance NK cytotoxicity in the context of refractory viral infections. A combination of NK cell activation by the IL-15 super-agonist ALT-803, anti-CD16-fusion antibodies or vaccines with antiretroviral therapy (ART) are in clinical studies to treat HIV infections [75,76]. NK cell therapies offer several advantages when compared to T cell-based approaches. First, NK cells do not rely on prior antigen sensitization. Second, they are less likely to trigger a cytokine-release syndrome. Third, they may have less toxic effects and do not drive excessive graft-versus-host disease [74,76]. Finally, NK cells can even come from non-autologous sources and may be beneficial for treating lymphopenic patients. Interestingly enough, links for ILC functions in systemic infections by HIV, Mtb, SARS-CoV-2 or bacterial sepsis are just emerging [77–82]. Hence, it is tempting to speculate that modulating ILC functions in certain infectious disease settings could be effective and support treatments of otherwise refractory diseases.

## Current and future challenges

In general, ILCs are primarily tissue-resident and organ-specific immune cells of very low abundance. Low cell numbers pose serious challenges for *in vivo* studies in either mice or humans, but such studies are pivotal for determining relevant *in vivo* functions. Correspondingly, an age-dependent decline in different ILC subsets may be relevant for health, but studies in aging mice or individuals are still scarce [30,64,80,83,84]. Moreover, human ILC1s and NK cells are very difficult to distinguish (Fig 1) [6,27], and unique, subset defining features remain elusive. One possibility is a high degree of plasticity as well as redundancy between these 2 subsets. This scenario mirrors Th subsets were transcriptional reprogramming or adaptive rewiring can derepress lineage-specific genes that determine T cell cytotoxicity [85]. In this case by down-regulating ThPOK and subsequent up-regulation of Runx3 along with the CBFβ-complexes [15,40,86]. Accordingly, Runx3 is also essential for development and maintenance of ILC1s and Nkp46^+^ILC3s, while ThPOK negatively regulates RORγt and IL23R expression [15,40,59]. In contrast, neither Runx3 nor the loss of ThPOK correlates with the induction of a cytotoxic effector program in ILCs, highlighting essential differences in regulation of these cell types. In mice, acquisition of an ILC1-associated transcriptional profile is rather linked to phenotypes associated with exhaustion [24,85,87]. In view of NK-cell-based therapies, it will be mandatory to understand the dynamics in human ILCs [85]. Other transcriptional regulators, in particular, histone deacetylase 1 (HDAC1) and HDAC2 are also potent regulators of cytotoxicity in T cells [88]. It will be exciting to further explore whether these chromatin modifiers can also contribute to the adaptive potential and plasticity of ILCs [89] similar to the situation in T cell lineages, where HDACs have been further associated with T helper subset defects in health and autoimmunity [88,90,91].

Further, transcriptional and phenotypical heterogeneities within ILC subsets are dynamically influenced by their immunological microenvironments. Thus, ILCs may reflect functional continua and appear as transition cell types [30,31]. Current strategies rely on negative selection of ILCs, sorted by the lack of lineage-defining surface markers and by the expression of IL-7Rα, CD90, and CD49a [2,92–95]. However, canonical ILC markers such as CD90 and IL-7R are subject to regulation and thus not constantly expressed on the surface of intestinal or pulmonary ILCs, respectively [31,32,96]. Moreover, the integrin CD49a may also show tissue-related variation [3,31]. Lineage-defining negative markers such as Ly6c may be in addition up-regulated following inflammatory challenges or in distinct subsets during steady state [18,19,31]. Categorization into canonical subsets can therefore create a bias in the interpretation of skyrocketing next-generation single-cell RNA-seq datasets. Hence, the analysis and clustering of single-cell data should be done in great detail and with caution, since interpretations should require at least some validation. Nonetheless, single-cell–based technologies still constitute an essential tool in studying small cell populations in general and ILCs particularly in an organ- or disease-specific context. Such technologies will without any doubt propel our understanding of the complex cellular interplay *in vivo* to further unravel relevant features of ILCs in disease.
